# Unraveling the role of male reproductive tract and haemolymph in cantharidin-exuding *Lydus trimaculatus* and *Mylabris variabilis* (Coleoptera: Meloidae): a comparative transcriptomics approach

**DOI:** 10.1186/s12864-021-08118-8

**Published:** 2021-11-08

**Authors:** Emiliano Fratini, Marco Salvemini, Fabrizio Lombardo, Maurizio Muzzi, Marco Molfini, Silvia Gisondi, Elia Roma, Veronica D’Ezio, Tiziana Persichini, Tecla Gasperi, Paolo Mariottini, Andrea Di Giulio, Marco Alberto Bologna, Manuela Cervelli, Emiliano Mancini

**Affiliations:** 1 Department of Sciences, University of Roma Tre, Rome, Italy; 2 Department of Biology, University of Naples Federico II, Naples, Italy; 3 Department of Public Health and Infectious Diseases, Sapienza University, Rome, Italy; 4 Department of Biology and Biotechnologies “Charles Darwin”, Sapienza University, Rome, Italy; 5 Natural History Museum of Denmark, Copenhagen, Denmark

**Keywords:** Cantharidin, Blister beetle, Toxic terpene, Reflex-bleeding, Autohaemorrhaging, Clotting, Detoxification, Defensive behaviour, Drug-delivery

## Abstract

**Background:**

Meloidae (blister beetles) are known to synthetize cantharidin (CA), a toxic and defensive terpene mainly stored in male accessory glands (MAG) and emitted outward through reflex-bleeding. Recent progresses in understanding CA biosynthesis and production organ(s) in Meloidae have been made, but the way in which self-protection is achieved from the hazardous accumulation and release of CA in blister beetles has been experimentally neglected. To provide hints on this pending question, a comparative de novo assembly transcriptomic approach was performed by targeting two tissues where CA is largely accumulated and regularly circulates in Meloidae: the male reproductive tract (MRT) and the haemolymph. Differential gene expression profiles in these tissues were examined in two blister beetle species, *Lydus trimaculatus* (Fabricius, 1775) (tribe Lyttini) and *Mylabris variabilis* (Pallas, 1781) (tribe Mylabrini). Upregulated transcripts were compared between the two species to identify conserved genes possibly involved in CA detoxification and transport.

**Results:**

Based on our results, we hypothesize that, to avoid auto-intoxication, ABC, MFS or other solute transporters might sequester purported glycosylated CA precursors into MAG, and lipocalins could bind CA and mitigate its reactivity when released into the haemolymph during the autohaemorrhaging response. We also found an over-representation in haemolymph of protein-domains related to coagulation and integument repairing mechanisms that likely reflects the need to limit fluid loss during reflex-bleeding.

**Conclusions:**

The de novo assembled transcriptomes of *L. trimaculatus* and *M. variabilis* here provided represent valuable genetic resources to further explore the mechanisms employed to cope with toxicity of CA in blister beetle tissues. These, if revealed, might help conceiving safe and effective drug-delivery approaches to enhance the use of CA in medicine.

**Supplementary Information:**

The online version contains supplementary material available at 10.1186/s12864-021-08118-8.

## Background

Insects use a wide array of defensive toxic compounds which may be produced autogenously by de novo synthesis or acquired by dietary sequestration of secondary compounds derived from plants, ingested prey, parental transfer, or endosymbionts. The strategy for using these toxins greatly differs among insects. ‘Reflex bleeding’ or ‘autohaemorrhaging’ [1] is the release of toxin-containing haemolymph from integumental ruptures when individuals are threatened or exposed to direct physical attack. Among all defensive strategies, this is probably the most physiologically costly and extreme to serve a harmful compound to a predator [2–4]. Among insects, Plecoptera, Orthoptera and Hemiptera include some taxa exhibiting reflex-bleeding defensive mechanisms [5]. In contrast, autohaemorrhaging is common in several species from different families of Coleoptera (e.g. Erotylidae, Lampyridae, Coccinellidae), but represent a typical trait of blister beetles (Meloidae) [6].

Meloidae is a widespread family including almost 3000 species [6–8] well-known for secreting cantharidin (C_10_H_12_O_4_; hereafter named CA), a toxic terpene. Blister beetles, if disturbed, naturally exude CA in yellowish oily haemolymph droplets from leg and antennal joints to defend themselves from predators. The concentration of the secreted CA vary from 0.03 to 0.79 mg for each gram of exuded haemolymph [9, 10], but largely depends on species, environmental and physiological conditions. Males produce more CA than females [9–15] and accumulate large amounts of this terpene in their reproductive organs and, particularly, in male accessory glands (MAG) [13, 16–19]. During mating, males transfer large quantities of CA to females [6, 20–23] which, in turn, use the received compound for protecting eggs from potential predators or parasites [13, 16, 21, 24].

CA is well-known in popular pharmacology for its traditional use as a sexual stimulant or antiphlogistic agent [6, 25–27]. However, CA is also a potent blistering compound causing many adverse effects after ingestion, such as severe damages to gastrointestinal, kidney and urinary tracts [6, 27]. The large use of CA in modern medicine is hindered by its extreme toxicity that renders its employment limited to the topical treatment of warts under strict legislative regulations [27, 28].

Recently, CA and its derivatives have regained popularity as alternative compounds for anti-cancer treatments [29–33]. Such a renewed interest has led to a growing literature devoted to reveal the molecular basis of CA de novo biosynthesis in blister beetles, focusing on species belonging to the (species-rich) Meloinae subfamily. Both gene-expression analyses of key-enzymes in different organs of *Epicauta sibirica* Pallas, 1773 (tribe Epicautini; all reported as *chinensis* Laporte, 1849) [18, 19, 34, 35] and a de novo transcriptomic approach comparing the relative abundance of transcripts in males *vs.* females in *Hycleus cichorii* (Linnaeus, 1767) (tribe Mylabrini; reported as *Mylabris*) [36] were coherent with previous studies indicating that: (i) CA may be synthetized via the mevalonate (MVA) pathway [37], (ii) farnesol may act as an intermediate [38–40], and (iii) a juvenile hormone (JH) metabolite could be involved in one of the latest biosynthetic steps [35, 37, 41].

However, there is still a gap of knowledge on some crucial steps of the CA biosynthetic pathway, as well as the specific site of its in vivo production. At first, the third pair of MAG was designated as the production site [16, 42], but recently, both high CA content and transcriptional level of the 3-hydroxy-3-methylglutary-CoA reductase (HMGCR, an essential enzyme of the MVA pathway) observed in the fat body of *E. sibirica* suggested that CA biosynthesis may more likely occur in this tissue [19].

McCormick et al. were the first to suggest that MAG represented the storage organ for CA and not the site of its biosynthesis [21, 41]: these authors hypothesized that the newly synthetized CA could first proceed to the haemolymph, from which it would have been removed by a ‘cantharidin kidney’ and stored in MAG [21]. Whatever the mechanism, a not-yet thoroughly addressed question regards the safe modes of CA circulation and storage. It is in fact still unclear how self-protection from the toxicity of a high CA content could be prompted in male reproductive organs, in which mechanisms able to inactivate or mild the action of the (incoming and accumulating) CA might be operating. Furthermore, still unidentified mechanisms should allow CA to be transported through the haemolymph from the fat body (or another production sites) to the storing organs, and then discharged outside the body without causing self-injuries.

A third, more general (and evolutionary) question concerns the identification of proteins in the haemolymph repertoire that should support such a metabolically expensive defensive strategy. In fact, yet highly effective, a haemolymph-mediated chemical defence requires a rapid renewal of water [43] and components of the immune system (e.g. alkaloids, haemocytes or antimicrobial peptides) after bleeding. Moreover, both efficient haemolymph-coagulating and integument repairing mechanisms are expected, as these should be essential requirements of autohaemorrhaging insects to limit haemolymph loss and guarantee their survival.

With this study, we attempted for the first time to shed light on modes of mobilization, storage, and deactivation of CA in blister beetles which shall prevent its adverse effects on organs/tissues where CA is stored and/or regularly circulates. To do so, we used a de novo assembly transcriptome approach and a differential gene expression analysis targeting the reproductive organs and exuded haemolymph (gathered during autohaemorrhaging) of males of two Meloinae species, i.e. *Lydus trimaculatus* (LT) [Fabricius, 1775 (tribe Lyttini)] and *Mylabris variabilis* (MV) [Pallas, 1781 (tribe Mylabrini)]. The choice of these two species was dictated by their: 1) proved ability to produce high titres of CA in natural conditions [10]; 2) relatively high abundance on field [10], crucial to acquire the amount of haemolymph necessary to gain reasonable RNA yields, and 3) ascription to two different tribes of Meloinae owning the proper level of phylogenetic divergence [6, 8] to observe a co-occurrence of abundantly expressed candidate detoxification genes. Besides, our approach allowed examining the expression levels of the thus-far-identified genes related to CA biosynthesis in male reproductive tracts (MRT) of LT and MV. We also provided a catalogue of over-represented protein families in haemolymph involved in the costly physiology of the autohaemorrhaging strategy.

## Results

### De novo assembly and annotation of LT and MV transcriptomes

Our final dataset consisted of 252,190,732 and 301,963,686 (50–150 bp) quality-filtered reads for LT and MV, respectively (Table 1). Both de novo assemblies received a high score of completeness (BUSCO ‘C’ values = ~ 97%) and produced for LT a total of 190,214 assembled transcripts, ranging from 401 to 31,991 bp, and corresponding to 111,614 Trinity unigenes, and for MV a total of 165,191 assembled transcripts, ranging from 401 to 26,915 bp, and corresponding to 94,199 Trinity unigenes (Table 1). We found a higher percentage of duplicated BUSCOs (D) in LT than in MV (Table 1). This can be considered a possible artifact originated by the high number of allelic variants (= high heterozygosity) in LT populations, being interpreted as multiple unigenes/isoforms. Since this did not affect our main conclusions, we decided to not de-replicate the obtained transcriptomes and maintain the whole information. By selecting the longest transcript isoform for each Trinity unigene cluster and using the Annocript pipeline (see Methods), we observed that 47.7% (LT) and 33.4% (MV) could be annotated with BLAST searches against UniRef [mostly from *Tribolium castaneum* Herbst, 1787; Coleoptera, Tenebrionidae], whereas 30.0% (LT) and 20.8% (MV) were annotated against Swiss-Prot [mostly from *Drosophila melanogaster* Fallén, 1823; Diptera, Drosophilidae]. Finally, 30.2% (LT) and 21.3% (MV) of the longest transcripts matched with functional protein domains in InterProScan with at least one match in one of the following repositories: SignalP, TMHMM, Pfam, SMART, Tigr and ProfileScan.

**Table 1:** De novo transcriptome assembly statistics, quality control and annotation for LT and MV

De novo assembly statistics (Trinity v2.3.1)	LT	MV
Number of reads (150 bp)	270,706,855	319,413,792
Number of ‘clean’ reads (50–150 bp)	252,190,732	301,963,686
Number of predicted ‘unigenes’	111,614	94,199
Number of predicted ‘transcripts’	190,214	165,191
Percent GC	33.09	32.72
Contig N50	1622	1659
Median contig length^a^	836	780
Average contig^a^	1219	1207
Total assembled bases^a^	231,947,268	199,377,335
Range of transcript length	401–31,991	401–26,915
Number of transcripts longer than 10,000	101	203
Number of transcripts longer than 5000	2215	2420
Number of transcripts longer than 3000	11,932	10,856
Number of transcripts longer than 2000	29,414	25,399
Number of transcripts longer than 1000	78,770	64,122
**Assembly quality control (Busco v3.0.1)**		
Complete (C)	1038 (97.4%)	1030 (96.6%)
Complete & Single Copy (S)	653	998
Complete & Duplicated (D)	385	32
Fragmented (F)	25	27
Missing (M)	3	9
**De novo transcriptome annotation (Annocript)**		
Number of ‘longest ORF’ sequences	111,614	94,199
Mean sequence length (and range) in bp	942 (401–31,991)	941 (401–26,915)
Average percentage of GC	34.25	33.47
UniRef ^b^	53,281	31,491
SwissProt^b^	33,546	19,670
Domains	33,801	20,067
Ribosomal RNAs	319	282
Transcripts with at least one blast result	53,694	31,892
Number of non-coding sequences ^c^	1322	1777
Gene Ontology	31,298	17,879

^a^ calculated based on all transcripts; ^b^ annotations generated based only on the longest unigene per transcript; ^c^ number of non-coding sequences were obtained with probability > 0.95 and maximum length of the ORF = 100

**Fig. 1:**
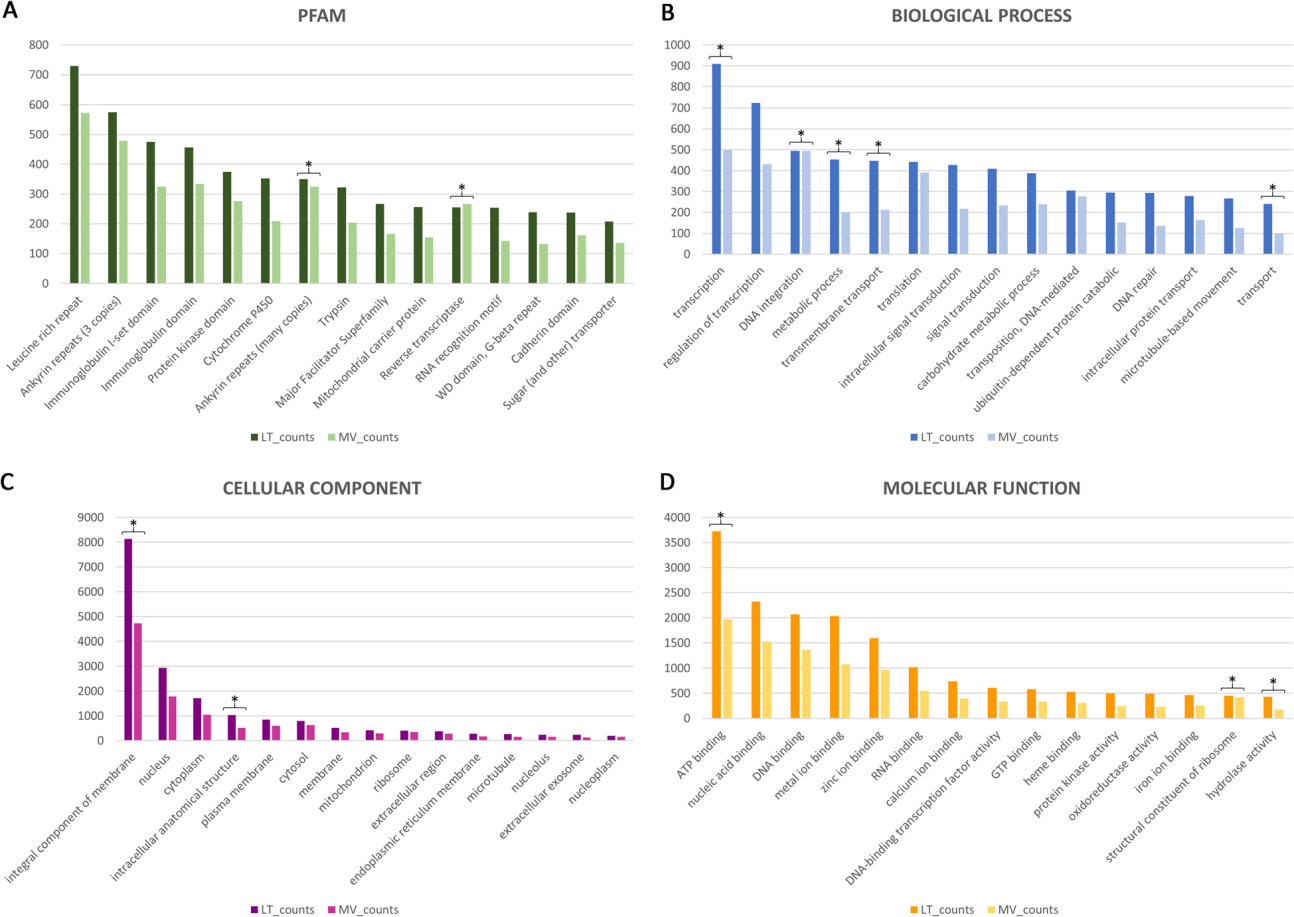
**Classification and relative abundance of top 15 protein families (Pfam) (A) and Gene Onthology (GO) terms in each category (B, C, D) in LT and MV transcriptomes*.*** Pfam and GO differentially expressed between species are marked with * (*p* < 0.05)

Detailed annotation information for individual LT and MV longest transcripts is available in Additional file 1. Classification and relative abundance of top 15 conserved Protein families (Pfam) and Gene Ontology (GO) terms are reported in Fig. 1. Residue motifs mediating protein-protein interactions and signalling, such as leucin rich (Pf13855) and ankyrin (Pf12796) repeats, as well as immunoglobulin-related domains (Pf07679, Pf13927) were among the most abundant Pfam in transcriptomes of both species (Fig. 1). ‘Protein kinase’ (Pf00069), ‘Cytochrome P450’ (Cyp450) (pf00067), ‘Reverse Transcriptase’ (pf00078) and ‘Trypsin’ (Pf00089) were also well-represented in both species (Fig. 1A), as well as protein families involved in movement of small solutes, such as ‘Major Facilitator Superfamily’ (Pf07690) and ‘Sugar (and other) transporters’ (Pf00083) (Fig. 1A). Overall, the relative abundances of GO were also coherent between the two analysed species. Among the most significant ‘GO-biological processes’, three DNA-related terms (‘transcription’, ‘regulation of transcription’, ‘DNA integration’) were highly frequent, but interestingly, also processes associated to transport (e.g. ‘transmembrane transport’ and ‘intracellular protein transport’) were highly represented (Fig. 1B). The most represented locations of gene products in cellular structures (‘GO-cellular components’) were, in order, ‘integral to membrane’, ‘nucleus’ and ‘cytoplasm’ (Fig. 1C). Finally, among ‘GO-molecular functions’, ‘ATP-binding’, ‘nucleic acid binding’, ‘DNA binding’ were the most recurrent, but, noteworthy, a number of other GO binding-related terms - such as metal-ion binding (zinc, calcium, iron) - were also rather abundant (Fig. 1D).

### Analysis of differentially expressed genes (DEGs)

**Fig. 2:**
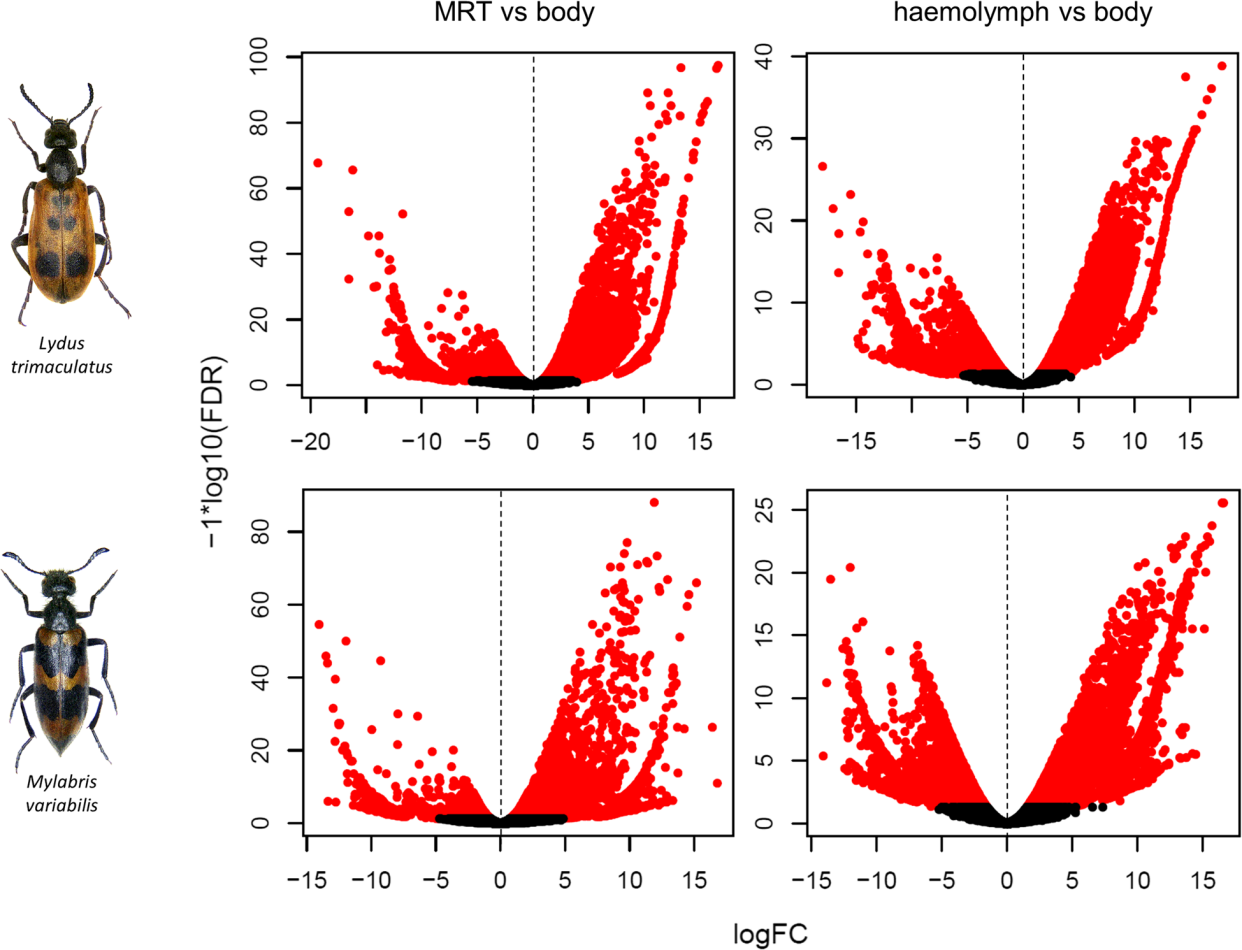
**Tissue-specific gene expression of MRT and haemolymph in LT and MV.** Volcano plots display the relative abundance levels of upregulated transcripts in MRT and haemolymph compared to the whole body. The x-axis represents the log2 of the expression ratio (FC = fold change) for each transcript (tissue specific logCPM: whole body logCPM, where CPM stands for Counts Per Million reads); the y-axis represents the log10 of the *p*-value corrected for the false discovery rate (FDR). Red dots represent differentially expressed transcripts with logFDR < 0.001 and at least 2-fold difference in logCPM; black dots if logFDR > 0.001. Negative logFC values indicate transcripts enhanced in the target tissue subsets, while positive values indicate transcripts upregulated in the whole body

Overall, the expression profiles observed for both targeted tissues in LT and MV were comparable in terms of relative abundance of differentially expressed transcripts (Fig. 2). Following a conservative approach (logFC > 2, FDR < 0.001), 1800 (LT) and 568 (MV) transcripts appeared upregulated in MRT when compared to the whole body, whereas 1275 (LT) and 1471 (MV) transcripts showed an enhanced expression in haemolymph (Fig. 2). Among the upregulated transcripts in these two tissues, ~ 15% did not display a significant similarity with known proteins in the non-redundant (nr) database after BLAST search. Comparison of DEGs between the two targeted tissues showed 5005 (LT) and 6139 (MV) transcripts with enhanced expression in MRT with respect to haemolymph, whereas 2186 (LT) and 2527 (MV) were upregulated in the haemolymph respect to MRT (Fig. 2). The complete list (and annotation) of DEGs (i.e. upregulated) in MRT and haemolymph of each species is reported in Additional file 2 (a list of downregulated genes in both tissues is available upon request).

Significantly over-represented GO terms in DEGs (when compared to the whole assemblies) are reported in Additional file 3. Among the most abundant ‘GO-biological processes’ already observed in the two whole transcriptomes, transmembrane and intra-cellular protein transportations were also specifically enriched in MRT and haemolymph of both species (Additional file 3). Enriched-GO shared between species in MRT and haemolymph are reported in Tables 2 and 3, respectively.

**Table 2:** Most significant (*p* < 0.05) GO terms enriched in MRT of both LT and MV. ALL = count number of all GO retrieved in transcriptome; UP = count number of enriched GO

			LT			MV		
	**ID**	**Description**	**ALL**	**UP**	***p***	**ALL**	**UP**	***p***
**Biological process**	GO:0036159	inner dynein arm assembly	8	6	8.48 × 10^−36^	9	7	4.66 × 10^−111^
	GO:0003341	cilium movement	25	9	6.19 × 10^−34^	16	8	4.36 × 10^−98^
	GO:0060271	cilium morphogenesis	53	10	5.85 × 10^−22^	29	5	2.38 × 10^−25^
	GO:0007018	microtubule-based movement	266	23	3.00 × 10^−22^	124	10	1.33 × 10^− 26^
	GO:0006974	response to DNA damage stimulus	47	5	1.84 × 10^−06^	35	5	1.50 × 10^−21^
	GO:0006355	regulation of transcription, DNA-dependent	724	18	6.60 × 10^− 03^	430	7	2.30 × 10^− 03^
**Molecular function**	GO:0008569	minus-end-directed microtubule motor activity	21	9	1.15 × 10^− 38^	18	8	1.82 × 10^−90^
	GO:0045503	dynein light chain binding	22	9	2.27 × 10^−37^	19	8	4.44 × 10^−87^
	GO:0051959	dynein light intermediate chain binding	23	9	3.74 × 10^−36^	20	8	6.21 × 10^−84^
	GO:0045505	dynein intermediate chain binding	27	9	5.80 × 10^−32^	22	8	2.85 × 10^−78^
	GO:0003777	microtubule motor activity	283	26	6.48 × 10^−27^	135	12	2.29 × 10^−35^
	GO:0005524	ATP binding	3724	122	1.77 × 10^−24^	1973	56	2.69 × 10^−45^
	GO:0008017	microtubule binding	236	20	5.43 × 10^−19^	147	5	1.32 × 10^−05^
	GO:0046872	metal ion binding	2030	53	6.82 × 10^−07^	1075	18	2.55 × 10^− 07^
	GO:0004842	ubiquitin-protein ligase activity	201	11	1.82 × 10^−06^	37	5	2.46 × 10^− 09^
	GO:0016887	ATPase activity	346	13	1.67 × 10^−04^	176	9	5.11 × 10^−15^
	GO:0031625	ubiquitin protein ligase binding	72	5	2.56 × 10^− 04^	87	5	1.59 × 10^−20^
	GO:0005509	calcium ion binding	737	20	1.22 × 10^−03^	394	11	2.72 × 10^−09^
	GO:0004674	protein serine/threonine kinase activity	324	11	1.82 × 10^− 03^	200	6	8.15 × 10^−06^
**Cellular component**	GO:0005874	microtubule	265	36	2.17 × 10^− 57^	154	14	2.18 × 10^−42^
	GO:0005929	cilium	33	12	2.88 × 10^−46^	31	12	2.54 × 10^−127^
	GO:0030286	dynein complex	108	15	1.40 × 10^−24^	42	9	1.82 × 10^−58^
	GO:0005737	cytoplasm	1719	72	1.04 × 10^−23^	1042	37	4.81 × 10^−40^
	GO:0016021	integral to membrane	8136	167	7.70 × 10^−09^	4727	60	3.49 × 10^−13^
	GO:0016020	membrane	520	21	2.39 × 10^−07^	336	14	1.34 × 10^−18^
	GO:0005634	nucleus	2937	65	2.97 × 10^−05^	1789	34	1.07 × 10^−15^
	GO:0005886	plasma membrane	843	25	5.15 × 10^− 05^	603	16	2.40 × 10^−12^
	GO:0005739	mitochondrion	419	12	7.14 × 10^−03^	285	8	5.31 × 10^− 07^

**Table 3:** Most significant (*p* < 0.05) GO terms enriched in haemolymph of both LT and MV. ALL = count number of all GO retrieved in transcriptome; UP = count number of enriched GO

			LT			MV		
	**ID**	**Description**	**ALL**	**UP**	***p***	**ALL**	**UP**	***p***
**Biological process**	GO:0006270	DNA-dependent DNA replication initiation	40	9	1.40 × 10^−38^	27	8	6.51 × 10^−31^
	GO:0007155	cell adhesion	98	12	5.98 × 10^− 30^	83	13	2.82 × 10^−29^
	GO:0051056	regulation of small GTPase med. Signal transduction	38	7	7.97 × 10^−25^	26	5	4.58 × 10^−13^
	GO:0007616	long-term memory	22	5	1.68 × 10^−20^	23	5	1.79 × 10^−14^
	GO:0007229	integrin-mediated signaling pathway	76	8	4.48 × 10^−17^	67	6	7.39 × 10^− 08^
	GO:0007411	axon guidance	64	7	1.92 × 10^−15^	70	6	1.57 × 10^−07^
	GO:0007165	signal transduction	408	14	1.92 × 10^−08^	234	13	1.31 × 10^−09^
	GO:0006979	response to oxidative stress	109	6	9.48 × 10^−07^	76	7	2.27 × 10^− 09^
	GO:0035556	intracellular signal transduction	427	12	1.07 × 10^−05^	217	7	5.02 × 10^−03^
	GO:0016192	vesicle-mediated transport	159	6	1.43 × 10^−04^	114	5	2.60 × 10^−3^
	GO:0016567	protein ubiquitination	142	5	1.12 × 10^−03^	75	5	5.49 × 10^−05^
	GO:0007049	cell cycle	145	5	1.34 × 10^−03^	87	7	3.53 × 10^−08^
	GO:0006260	DNA replication	182	5	7.26 × 10^− 03^	74	7	1.27 × 10^−09^
**Molecular function**	GO:0005096	GTPase activator activity	183	14	1.19 × 10^−21^	112	12	1.58 × 10^−18^
	GO:0003678	DNA helicase activity	76	8	4.48 × 10^−17^	55	7	8.04 × 10^−13^
	GO:0051015	actin filament binding	120	10	5.96 × 10^− 17^	70	7	3.65 × 10^−10^
	GO:0046872	metal ion binding	2030	50	3.13 × 10^−16^	1075	34	4.23 × 10^− 10^
	GO:0005524	ATP binding	3724	70	3.80 × 10^− 13^	1973	48	3.15 × 10^−08^
	GO:0003779	actin binding	234	11	5.71 × 10^− 10^	147	14	4.72 × 10^−19^
	GO:0005200	structural constituent of cytoskeleton	56	5	4.92 × 10^−09^	31	6	5.92 × 10^− 16^
	GO:0005525	GTP binding	581	16	4.92 × 10^−07^	331	11	2.49 × 10^− 03^
	GO:0003700	sequence-specific DNA bind. Transcr. factor activity	605	17	1.27 × 10^− 07^	335	9	7.62 × 10^− 03^
	GO:0003723	RNA binding	1017	22	2.51 × 10^−06^	545	18	2.57 × 10^− 06^
	GO:0042802	identical protein binding	123	6	5.74 × 10^− 06^	87	6	4.55 × 10^− 06^
	GO:0005509	calcium ion binding	737	17	1.07 × 10^−05^	394	14	9.96 × 10^− 06^
	GO:0003924	GTPase activity	426	11	9.50 × 10^− 05^	249	10	3.91 × 10^− 05^
	GO:0004930	G-protein coupled receptor activity	258	7	1.37 × 10^−03^	159	10	9.57 × 10^− 09^
	GO:0003677	DNA binding	2066	29	2.17 × 10^− 03^	1362	25	8.86 × 10^− 03^
	GO:0008017	microtubule binding	236	6	5.41 × 10^− 03^	147	7	1.07 × 10^− 03^
**Cellular component**	GO:0042555	MCM complex	29	8	3.05 × 10^− 40^	18	8	5.19 × 10^− 42^
	GO:0031594	neuromuscular junction	14	5	3.98 × 10^− 29^	16	6	4.09 × 10^− 27^
	GO:0005886	plasma membrane	843	35	1.63 × 10^− 25^	603	41	7.88 × 10^− 37^
	GO:0005737	cytoplasm	1719	47	2.89 × 10^−18^	1042	44	1.91 × 10^−20^
	GO:0005925	focal adhesion	89	8	1.25 × 10^− 14^	34	7	8.30 × 10^− 20^
	GO:0005634	nucleus	2937	54	6.24 × 10^− 10^	1789	48	4.47 × 10^− 10^
	GO:0031012	extracellular matrix	76	6	1.18 × 10^− 09^	40	5	5.72 × 10^− 09^
	GO:0016021	integral to membrane	8136	107	1.68 × 10^− 07^	4727	125	8.67 × 10^− 24^
	GO:0000139	Golgi membrane	177	8	3.97 × 10^− 07^	112	5	2.27 × 10^− 03^
	GO:0005887	integral to plasma membrane	164	7	6.04 × 10^− 06^	122	8	1.79 × 10^− 07^
	GO:0016020	membrane	520	13	3.52 × 10^− 05^	336	14	4.05 × 10^− 07^
	GO:0005829	cytosol	788	17	3.90 × 10^− 05^	620	22	2.45 × 10^− 08^
	GO:0005622	intracellular	1029	19	2.52 × 10^− 04^	512	16	2.75 × 10^− 05^
	GO:0005576	extracellular region	380	9	1.18 × 10^− 03^	273	8	6.03 × 10–03
	GO:0005856	cytoskeleton	154	5	2.18 × 10^− 03^	99	6	2.53 × 10^− 05^
	GO:0005874	microtubule	265	6	1.22 × 10^− 02^	154	6	2.37 × 10^− 03^

In MRT, dynein, cilium- and microtubule-related GO terms (likely associated with cytoskeletal dynamics in spermatogenesis) were the most over-represented, whereas others were related to ubiquitination, transcription regulation and phosphorylation (i.e. ‘serine/threonine kinase’) (Table 2). In haemolymph, most of shared enriched GO terms pertained to DNA replication, cytoskeleton, cell adhesion, actin binding (probably related to clotting), signal transduction and vesicle mediated-transport, but responses to oxidative stress and activities of G-protein coupled receptors were also recorded (Table 3). MRT- and haemolymph-specific Pfam upregulated in each species (adj. *p* < 0.05) are reported in Additional file 4. The subsets of Pfam (adj. *p* < 0.05) shared between LT and MV are reported in Table 4 (MRT) and Table 5 (haemolymph), respectively.

**Table 4:** Pfam shared and enriched in MRT of both LT and MV (adj *p* < 0.05). ALL = count number of all Pfam retrieved in transcriptome; UP = count number of enriched Pfam

		LT			MV			
Pfam ID	Description	ALL	UP	adj. ***p***	ALL	UP	adj. ***p***	Putative function
pf12774	Hydrolytic ATP bind. Site of dynein motor region D1	25	10	2.9 × 10^−11^	16	7	2.54 × 10^−14^	dynein motor
pf03028	Dynein heavy chain and region D6 of dynein motor	39	12	1.09 × 10^−10^	27	8	7.04 × 10^− 12^	dynein motor
pf15921	Coiled-coil domain-containing protein 158	62	15	1.70 × 10^−10^	46	13	9.71 × 10^− 19^	sperm regulation
pf12780	P-loop containing dynein motor region D4	22	9	2.05 × 10^−10^	14	7	8.62 × 10^− 16^	dynein motor
pf08393	Dynein heavy chain, N-terminal region 2	31	10	2.7 × 10^−09^	23	9	5.54 × 10^− 17^	dynein motor
pf12777	Microtubule-binding stalk of dynein motor	24	8	1.77 × 10^− 07^	13	7	1.29 × 10^− 16^	dynein motor
pf12775	P-loop containing dynein motor region D3	19	7	3.69 × 10^− 07^	14	6	7.56 × 10^− 12^	dynein motor
pf12781	ATP-binding dynein motor region D5	19	7	3.69 × 10^− 07^	15	7	6.19 × 10^− 17^	dynein motor
pf07728	AAA domain (dynein-related subfamily)	67	10	1.72 × 10^− 03^	34	8	1.67 × 10^− 09^	dynein motor
pf08385	Dynein heavy chain, N-terminal region 1	21	5	5.81 × 10^− 03^	18	5	1.83 × 10^−6^	dynein motor
pf13868	Trichohyalin-plectin-homology domain	8	3	1.68 × 10^−02^	3	3	2.73 × 10^− 10^	structural organization
pf00412	LIM domain	66	8	4.58 × 10^−02^	69	7	6.87 × 10^−03^	regulatory

**Table 5:** Pfam shared and enriched in haemolymph of both LT and MV (adj *p* < 0.05). ALL = count number of all Pfam retrieved in transcriptome; UP = count number of enriched Pfam

		LT			MV			
Pfam ID	Description	ALL	UP	adj. ***p***	ALL	UP	adj. ***p***	Putative function
pf00630	Filamin/ABP280 repeat	64	23	6.57 × 10^−40^	44	19	4.20 × 10^− 22^	cellular immune response
pf00191	Annexin	42	13	1.27 × 10^− 18^	23	11	1.72 × 10^− 13^	protein trafficking
pf00493	MCM2/3/5 family	29	9	2.77 × 10^− 12^	20	8	5.32 × 10^− 08^	hematopoiesis (DNA replication)
pf02145	Rap/ran-GAP	9	5	4.45 × 10^−10^	10	4	1.40 × 10^− 3^	regulatory
pf00168	C2 domain	110	15	2.41 × 10^−08^	95	15	3.77 × 10^− 05^	signal transduction
pf14551	MCM N-terminal domain	12	5	4.59 × 10^− 08^	10	6	4.28 × 10^− 08^	hematopoiesis (DNA replication)
pf00372	Hemocyanin, copper containing domain	7	4	7.45 × 10^− 08^	4	4	5.27 × 10^− 07^	clotting cross-link (prophenoloxidase)
pf06268	Fascin domain	3	3	9.79 × 10^− 08^	3	3	7.45 × 10^− 05^	Actin-bundling protein Singed
pf17207	MCM OB domain	22	6	7.12 × 10^− 07^	9	6	9.28 × 10^−09^	hematopoiesis (DNA replication)
pf00806	Pumilio-family RNA binding repeat	4	3	1.67 × 10^− 06^	3	3	7.45 × 10^−5^	mRNA regulation
pf08742	C8 domain	17	5	5.58 × 10^−06^	9	9	1.54 × 10^− 18^	clotting component
pf00094	von Willebrand factor type D domain	27	6	1.32 × 10^− 05^	13	11	8.99 × 10^− 21^	clotting component
pf00595	PDZ domain	136	12	1.71 × 10^− 03^	86	11	1.06 × 10^− 02^	protein-protein interaction/signal transduction
pf00435	Spectrin repeat	71	8	1.93 × 10^− 03^	71	27	4.40 × 10^−28^	protein trafficking
pf01290	Thymosin beta-4 family	3	2	1.93 × 10^− 03^	3	3	7.45 × 10^− 05^	tissue repair/regulation of hematopoiesis
pf03723	Hemocyanin, ig-like domain	3	2	1.93 × 10^− 03^	6	4	2.37 × 10^−05^	clotting cross-link (prophenoloxidase)
pf00052	Laminin B (Domain IV)	12	3	8.07 × 10^−03^	7	4	7.45 × 10^−05^	immunity against bacterial infections
pf02210	Laminin G 2	70	7	1.55 × 10^− 02^	54	10	2.69 × 10^−03^	immunity against bacterial infections
pf01826	Trypsin Inhibitor like cysteine rich domain	14	3	1.74 × 10^− 02^	12	4	5.37 × 10^−03^	Associated with hemocytin
pf00054	Laminin G domain	57	6	2.13 × 10^−02^	40	10	3.23 × 10^−06^	immunity against bacterial infections
pf00788	Ras association (RalGDS/AF-6) domain	15	3	2.47 × 10^−02^	12	5	7.45 × 10^−05^	signal transduction
pf00169	Pleckstrin homology (PH) domain	60	6	2.98 × 10^−02^	42	7	1.51 × 10^− 02^	signal transduction
pf16077	Spaetzle	29	4	3.32 × 10^−02^	22	6	5.74 × 10^− 04^	innate immunity

In MRT most of shared Pfam were related to motor functions of cilia and flagella, microtubule dimerization and transport of organelles and vesicles along microtubules (i.e. dynein-related subfamilies, e.g. pf12774 and pf03028; Table 4). Other shared and highly enriched domains were possibly involved in structural and/or regulation processes (i.e., pf13868; pf00412; pf15921; Table 4). Noteworthily, some significantly enriched domains in MRT of LT (but also detected in MV, yet with *p* > 0.05) were related to protein phosphorylation (pf00069) or, possibly, detoxification, such as glutathione synthases (pf03917) and zinc carboxypeptidases (pf00246) (Additional file 4: Table S11-S12). Additional species-specific protein families were linked to other processes, such as aminopeptidases (pf00883, pf02789) in LT or carboxylases (pf05090) and hydrolases (pf00723) in MV (Additional file 4: Table S11-S12). In haemolymph, most of shared enriched-Pfam were related to actin cross-linking proteins (e.g. pf00630, pf06268), vesicle transport (e.g. pf00191) or cell/membrane adhesion (e.g. pf00052) (Table 5). Other shared domains were related to oxygen transport (hemocyanin; pf03723), DNA replication processes (Minichromosome maintenance; pf00493, pf14551, pf17207), RNA binding (Pumilio family; pf00806) and lipid-binding (e.g. StAR-related lipid-transfer; pf01852) (Table 5). Other, but more species-specific domains were still related to the above-mentioned functions (Additional file 4). Interestingly, a subset of Pfam were clearly related to innate immunity responses (e.g. Spätzle: pf16077 and CUB: pf00431) with some domains more specifically involved in coagulation (haemolectins/mucins; e.g. C8: pf08742; von Willebrand factor: pf00094; Mucin2_WxxW: pf13330) and, possibly, in the formation of the viscous (oily) haemolymph-exudate (Table 5; Additional file 4: Table S13-S14).

### Genes putatively involved in CA biosynthesis

**Fig. 3:**
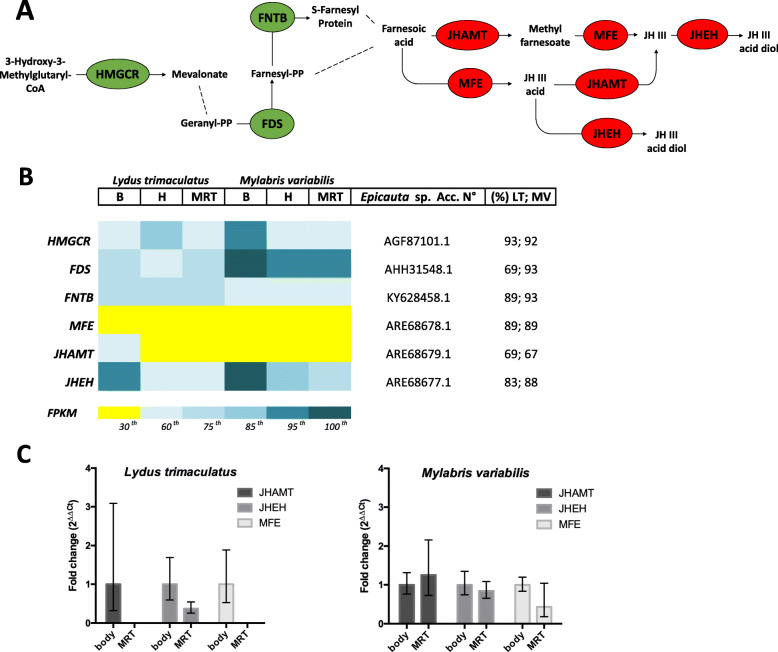
**Pathway and expression levels (Heat-Map and RT-qPCR) of genes putatively involved in CA biosynthesis in LT and MV*.*** A) The diagram shows a recap of the pathways involved in the synthesis and degradation of JH, highlighting the enzymes whose transcripts were here selected and examined. Green ellipses represent the enzymes of the upstream mevalonate and terpenoid backbone pathways, the red ones indicate the enzymes specific for the downstream JH pathway. B) Colours in the Heat-Map were assigned according to percentiles, with yellow referring to the lowest FPKM values (below 30th percentile) and dark blue to the FPKM highest values (above 95th percentile). Identity percentages of tBLASTn using *Epicauta* protein sequences (as in GenBank Acc. N.) as a query are reported in the last column. B =  body, MRT =  male reproductive tract, H =  haemolymph. C) The graphs show RT-qPCR results expressed as fold change (2^-ΔΔCt^) for three enzymes of the JH pathway, i.e. JHAMT, JHEH and MFE in LT and MV, respectively

We searched in both transcriptomes for unigenes related to the MVA pathway and trans-farnesol branch (“terpenoid backbone biosynthesis”, KEGG map 009000), as well as for enzymes involved in the JH biosynthesis (“insect hormone biosynthesis”, KEGG map 00981). In these pathways, we selected genes previously reported as related with CA content in blister beetles (i.e. in *Epicauta* and/or *Hycleus*, whose sequence data were available); further selection was driven by protein sequence homology with the ones from *Epicauta*, which allowed recognising six transcripts with high similarity (70–93%). Those pathways are summarized in Fig. 3A, in which the six genes selected are highlighted. Intensity of expression, as revealed by FPKM counts in transcriptome analyses, is reported in Fig. 3B (a complete list of transcripts emerging from BLAST searches on LT and MV transcriptomes can be found in Additional file 5: Table S15). MVA enzymes expressed in LT and MV transcriptomes included acetyl-CoA C-acetyltransferase (atoB; 2.3.1.9), hydroxyl-methylglutaryl-CoA synthase (HMGCS; 2.3.3.10), hydroxyl-methylglutaryl-CoA reductase (HMGCR; 1.1.1.34), mevalonate kinase (mvaK1; 2.7.1.36), phosphomevalonate kinase (mvaK2; 2.7.4.2) and diphosphomevalonate decarboxylase (MVD; 4.1.1.33). Transcripts encoding these enzymes all showed high identity (~ 60–80%) in both species with *Tribolium* protein putative orthologs (except for mvaK1 showing a 44% of similarity, and, thus, not further considered), but none was significantly upregulated in MRT or haemolymph in respect to the whole body. Among these genes, HMGCR - a rate-limiting enzyme in MVA pathway and crucial for CA synthesis [34] - was downregulated in MRT (Fig. 3B). We also searched for transcripts related to the farnesyl diphosphate synthase (FDS; 2.5.1.1, 2.5.1.10), a key enzyme in farnesol metabolism, but also intervening in the formation of the geranyl diphosphate, a precursor of sesquiterpenoids, triterpenoids and monoterpenoids. This gene, moderately abundant in the whole body of both species (~30–45 FPKM), was significantly downregulated in MRT and haemolymph of LT and MV (Fig. 3B). Among genes belonging to the branched chain of trans-farnesol (a potential precursor of CA), we identified NADP+-dependent farnesol dehydrogenase (FOHSDR: 1.1.1.216), protein-S-isoprenylcysteine O-methyltransferase (ICMT: 2.1.1.100), STE24 endopeptidase (STE24: 3.4.24.84) and protein farnesyltransferase subunit beta (FNTB: 2.5.1.58). All transcripts putatively encoding these genes were expressed but downregulated in MRT or haemolymph respect to whole body (Additional file 5: Table S15; Fig. 3B). Since a metabolite of JH could constitute a precursor of CA, we also investigated genes involved in JH biosynthetic pathway. Transcriptomic data showed that transcripts of two key enzymes responsible for the de novo synthesis of JH, i.e. methyl farnesoate epoxidase (MFE: 1.14.14.127; 1.14.14.128) and JH acid O-methyltransferase JH-III synthase (JHAMT: 2.1.1.325), were expressed at very low levels in both species (Fig. 3B). On the contrary, the juvenile hormone epoxide hydrolase (JHEH), involved in the degradation of JH, was abundant in whole bodies (~ 48–97 FPKM), though significantly downregulated in MRT of both species (Fig. 3B). Moreover, searching for members of the Cyp450 family, we identified unigenes showing 75% similarity with CYP4BM1, whose high expression level was demonstrated to be related to CA production [19]. These transcripts were significantly downregulated in MRT of both species as compared to the whole body (Additional file 5: Table S15) (as well as all transcripts showing a similarity with CYP4C7, a sesquiterpenoid omega-hydroxylase degrading JH III [44]).

Finally, we did not detect specific transcripts associated to genes of the sesquiterpenoid, triterpenoid or monoterpenoid biosynthetic pathways. Also, we did not find transcripts possibly encoding enzymes catalysing cyclization reactions (as would be required for the transformation of a linear sesquiterpenoid into the tricyclo-decane structure of CA), such as the *trans*-isoprenyl diphosphate synthase involved in cyclization of 8-oxogeranial to build the iridoid ring scaffold in flea beetles (Coleoptera: Chrysomelidae: Alticini) [45]. Rather, we identified transcripts referable to two key enzymes involved in the formation of terpenoid-quinone biosynthesis, such as the geranylgeranyl pyrophosphate synthase (2.5.1.1 2.5.1.10 2.5.1.29) and the 1,4-dihydroxy-2-naphthoate octaprenyltransferase (2.5.1.74), which, however, were not differentially expressed in MRT or haemolymph of both species as well.

### Identification of candidate genes related to CA detoxification and transport

**Fig. 4:**
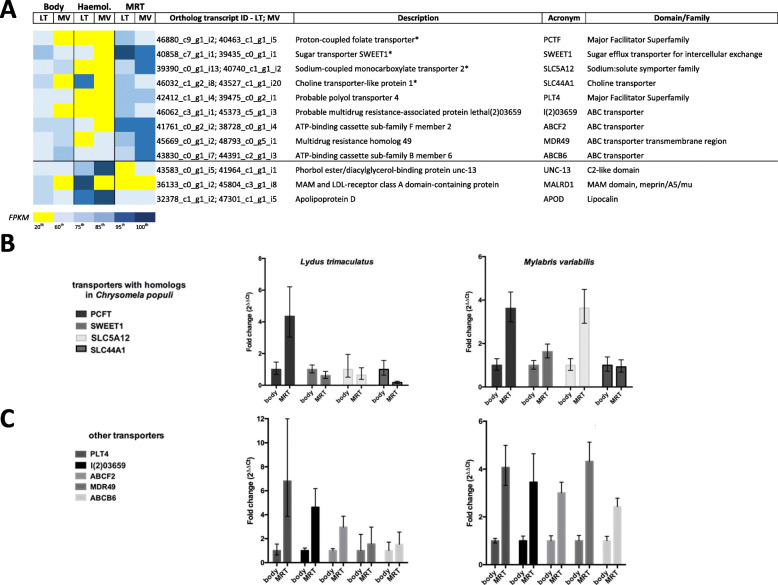
**Expression levels (Heat-Map and RT-qPCR) of genes putatively involved in sequestration mechanisms related to CA detoxification in LT and MV.** A) Colours in the Heat-Map were assigned according to percentiles, with yellow referring to the lowest FPKM values (below 20th percentile) and dark blue to the FPKM highest values (above 95th percentile). Transcripts showing identities with those involved in salicin sequestration in *Chrysomela populi* (see Discussion) are marked with (*). B) The graphs show RT-qPCR results expressed as fold change (2^-ΔΔCt^) for transcripts showing similarities to the ones involved in salicin sequestration in *Chrysomela populi*, i.e. PCFT, SWEET1, SLC5A12 and SLC44A1 in LT and MV, respectively. C) The graphs show RT-qPCR results expressed as fold change (2^-ΔΔCt^) for other transcripts possibly involved in CA transport, i.e. PLT4, l(2)03659, ABCF2, MDR49 and ABCB6 in LT and MV, respectively

The most conservative approach (logFC> 2, FDR < 0.001) identified 157 and 70 orthologous transcripts upregulated in both species in MRT and haemolymph, respectively. By relaxing the threshold (i.e., FDR < 0.01) the number of orthologous transcripts between the two species raised to 482 (MRT) and 283 (haemolymph), respectively. By applying the less conservative threshold, upregulated orthologous unigenes potentially involved in CA transport and/or detoxification were identified (Fig. 4A; Additional file 5: Table S16). Among them, two members belonging to ‘Major Facilitator Superfamily’, four to ‘ABC transporters’ and three to proteins involved in mobilization of small solutes (i.e. three sugar transporters, one sodium:solute symporter and one choline transporter) were identified in MRT, whereas three lipid-binding proteins were detected in the haemolymph (Fig. 4A). No unigenes coding for members of the most common families of detoxification enzymes (e.g. CypP450s, glutathione S-transferases) were identified.

### Reverse transcription-quantitative PCR (RT-qPCR) validation

Abundance of transcripts emerging from transcriptomic analyses has been confirmed by RT-qPCR by examining 12 transcripts differentially modulated in MRT and total body. We first examined transcripts coding for both JHAMT and MFE, two key enzymes of the latest steps of JH biosynthetic pathway, and for JHEH, a JH-degrading enzyme putatively involved in the production of a CA precursor. All three unigenes did not show any upregulation in MRT (Fig. 3C). Then, we analysed 4 transcripts coding for proteins whose possible homologs in *Chrysomela populi* Linnaeus, 1758 (Coleoptera, Chrysomelidae) are involved in transport of a plant-derived glycoside (salicin) from haemolymph to specific defensive glands (see Discussion): (i) proton-coupled folate transporter (PCFT), (ii) sugar transporter (SWEET1), (iii) sodium-coupled monocarboxylate transporter 2 (SLC5A12) and (iv) choline transporter-like protein 1 (SLC44A1) (Fig. 4A). Two of them, PCFT and SLC5A12 showed a strong differential expression in MRT compared to whole body (Fig. 4B). Finally, 5 transcripts encoding other putative transporters, chosen for ligand similarity, were analysed for their possible involvement in CA sequestration: i) probable polyol transporter 4 (PLT4), ii) probable multidrug resistance-associated protein lethal (2)03659 [l (2)03659], iii) ATP-binding cassette sub-family F member 2 (ABCF2), iv) multidrug resistance protein homolog 49 (MDR49), v) ATP-binding cassette sub-family B member 6 mitochondrial (ABCB6) (Fig. 4A). All of them exhibited a higher expression in MRT compared to whole body, particularly in MV (Fig. 4C).

**Fig. 5:**
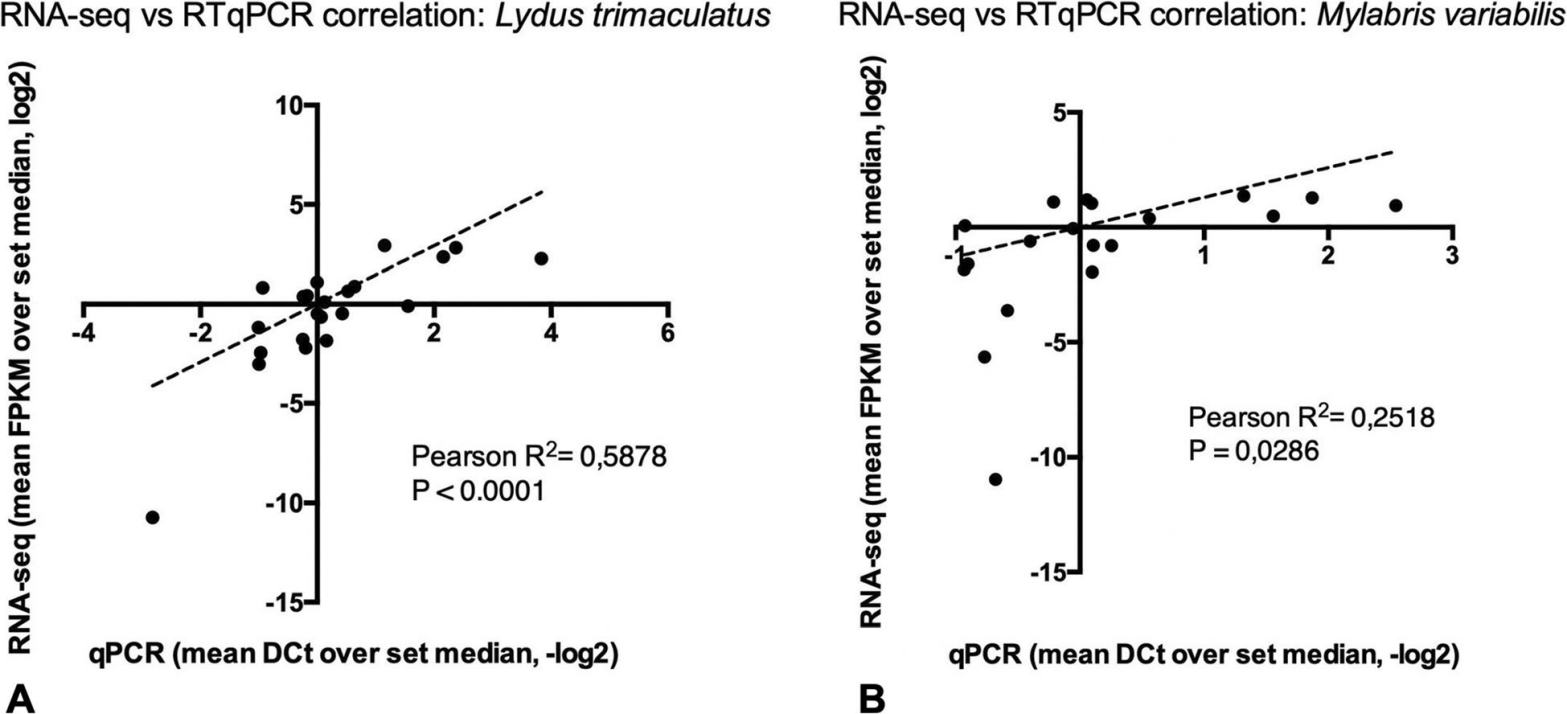
**RT-qPCR validation.** Correlation between transcriptional abundance of 11 genes (in LT) and 10 genes (in MV) in both whole body and dissected MRT samples, as revealed by RT-qPCR and RNA-seq. Level of abundance is defined as the ratio between each sample value over the group median (mean FPKM and mean ΔCt for RNA-seq and RT-qPCR data, respectively) in both RT-qPCR and RNA-seq approaches. For both techniques, statistical evaluation throughout Pearson test was performed and relative results are reported in the figure insets

The validation analysis was performed using independent, triplicate RNA samples extracted from whole bodies and MRT. Correlation analyses indicate statistically significant (R^2^ = 0.5878; *p* < 0.0001, for LT; R^2^ = 0.2518; *p* < 0.0286, for MV) linear relationships between RNA-seq and RT-qPCR results in both species (Fig. 5). This result strongly supports the transcriptional abundance profiles revealed by RNA-seq analyses, in terms of both absolute transcript abundance and relative fold-change differences of transcripts among tissue and samples.

## Discussion

The present study produced a de novo assembly of male transcriptomes of LT and MV, two blister beetle species (Meloidae) belonging to the Meloinae subfamily. A high-throughput characterization of the upregulated portion of transcriptome in MRT and haemolymph of CA-exuding blister beetles is here provided for the first time. The two target tissues are essential in understanding the biological defence mechanisms in Meloidae due to their close interaction with CA; in fact, the MRT corresponds to a specialized body compartment where CA is accumulated in large quantities (and from which it is released upon mating), whereas haemolymph represents a system of internal vehiculation and outward emission of CA under stressful conditions. The most relevant aspects emerging from our comparative analysis are discussed below.

### High quality of LT and MV de novo transcriptome assembly and conservation of gene expression patterns

A high-quality de novo transcriptome assembly, supported by the high percentage of (BUSCO) completeness (~ 97%) in terms of the expected gene content, was produced for LT and MV (Table 1). The overall size of the assembled transcriptomes (~ 200 Mb), as well as the number of retrieved transcripts (~180,000 on average), were similar in the two target Meloinae species (Table 1). These results likely mirror a comparable amplitude in the two species of the gene repertoire active in males, as it is expected in (relatively) phylogenetically close taxa having analogous physiological, ecological, and behavioural traits [6, 7]. It is also worth noticing that the most frequent protein domains and functional categories to whom the annotated transcripts were ascribed were nearly the same for both beetles (Fig. 1) and partially overlapped with those recovered in *Hycleus* [36]. These were basically related to nucleic acid synthesis and regulation, signal transduction, cytoskeleton-mediated transport, and metabolite transfer, and might be coherent with the activation of metabolic processes of sexually mature male insects. Such a convergence may also depend on the fact that males were in both cases collected from field after their emerging period, maintained in laboratory at steady rearing conditions and kept nourished on their natural host plants. The observed resemblance, however, remains partly speculative, since, as in other non-model insects [46], nearly 50–65% of recovered transcripts were unannotated. This however likely constitutes a valuable portion of transcriptomes to disclose possible underlying differences, and, above all, to identify proteins conferring peculiar adaptations to blister beetles. Remarkably, we also observed that the number of transcripts specifically upregulated in MRT and haemolymph of both LT and MV was comparable (Fig. 2). This outcome, again, might suggest a conservation of transcriptomic responses in these two tissues to provide fundamental functions in both species, almost certainly including those related to CA metabolism.

### Gene expression and role of the male reproductive system in CA biosynthesis

Overall, the transcriptome of MRT of both species encompassed a large set of protein domains whose enrichment was expected based on the fundamental role that these organs play in insect reproduction. Dynein, kinesin, microtubule-associated domains were the most abundant protein families, plausibly involved in mobilization of sperms, but also of secretory vesicles (and of their associated lipid and protein-based content) that were observed to replenish MAG [47]. Many other domains were associated to germ-cell proliferation or, more in general, to those developmental processes ongoing in reproductive organs of male insects, usually mediated by phosphorylation, nucleic acid regulation, ubiquitination and induction of many signalling cascades (as in *T. castaneum*) [48]. Among the latter, we observed many kinases, as well as a group of neuralized-like ubiquitin ligases known to play roles in Notch pathway-mediated cell fate decisions during development [49]. Furthermore, we also recorded carboxypeptidases that, along with other peptidases (e.g. aminopeptidases and trypsins in LT), are known to play a role in various aspects of male reproduction [50, 51].

Besides their reproductive functions, MRT, and particularly MAG, store large quantities of CA [13, 16–19]. However, MAG were recently disregarded as the site of CA production, as the enzymes involved in the earlier steps of CA biosynthesis were highly expressed in other organs [19, 52]. In fact, HGMCR - a key enzyme of the MVA pathway that directly alter the amount of downstream CA biogenesis in blister beetles [34] - showed a 60-fold higher expression in the CA-replenished fat body of *E. sibirica* than in other tissues [19]. Additionally, FDS, a key enzyme of the isoprenoid pathway synthesizing the farnesyl diphosphate (FPP), a precursor of various essential isoprenoid end-products, was hitherto found upregulated in the alimentary canal of males of *H. cichorii* [52]. Coherently with these previous observations, HMGCR and FDS (as well as unigenes encoding enzymes of the farnesol/farnesal-related branch of the terpenoid synthetic pathway; Additional file 5: Table S15) were both downregulated in MRT of LT and MV (Fig. 3B; Additional file 5: Table S15). Remarkably, among all, FDS appeared highly expressed in whole bodies of both species (and particularly in MV; see Fig. 3 and Additional file 5: Table S15). Hence, as already suggested [19, 52], FDS represents a plausible candidate gene for the synthesis of CA (along with other terpenoids) in blister beetles.

It has also been hypothesized that CA may originate from a JH-derived metabolite [35, 40]. In fact, silencing of two enzymes of the JH pathway, i.e. MFE and JHEH (but not JHAMT) caused a significant decrease in CA concentration in 3-to-7 days post-mated males of *E. sibirica* [35]. Hence, the JH III acid diol (a degradation product of JH III; Fig. 3A) was hypothesized as a potential precursor of CA [35]. JHs are sesquiterpenoids normally synthesized by corpora allata and released into the haemolymph where they regulate larval growth and metamorphosis [53]. In adults, JHs stimulate vitellogenesis and egg production in females [54] and orchestrate both the courtship behaviour and maturation of accessory glands in males [55, 56]. Since MRT of blister beetles can be reached by JH through the haemolymph, a degradation step of this hormone favouring a downstream CA synthesis could theoretically take place in this organ. Transcripts encoding two enzymes involved in the de novo synthesis of JH, i.e. MFE and JHAMT, were overall scarcely expressed (FPKM < 1) in LT and MV (Fig. 3B). This could imply that the JH titre at time of collection on field of (haemolymph-exuding) LT and MV was already adequate to ensure both the onset of reproductive maturation of males and CA renewal in their bodies. On the contrary, the mRNA of the JH-degrading enzyme JHEH was abundantly expressed in whole bodies of males of both species (Fig. 3B). The active conversion of JH into JH acid diol promoted by JHEH might serve both to suppress JH signalling and response [57] and supply JH-related metabolites to sustain CA biosynthesis. Since both our RNA-seq and RT-qPCR analyses confirmed that JHEH was significantly downregulated in MRT of both species (Fig. 3B-C), this might indicate that the JH-degrading route leading to CA was not expressed very highly in MRT at the time the samples were captured and dissected (i.e. CA may not be synthesized during the actual defence response, but accumulated before emission) and/or that it may more likely take place elsewhere in blister beetle body. Both options are plausible as in males of *E. sibirica* the highest transcript level of JHEH was observed 5th day after mating - in a phase of high CA productivity - and more significantly in the fat body [35].

Despite our data are coherent with previous findings downsizing the importance of MAG in the biosynthesis of CA, these might still constitutively express a key enzyme converting a linear terpenoid precursor into the typical tricyclo-decane structure of CA in one of the very latest steps of the biosynthetic pathway. Although we failed to detect transcripts pertaining to *IDS*-related terpene synthases - a family of enzymes known to perform terpene cyclization in insects [45] – some other overlooked biochemical processes converting a final metabolite delivered through the haemolymph, could eventually take place in MAG and contribute to accumulate CA in this organ. To conclude, it is worth to remark that tailored genomic analyses on fat bodies will be necessary to possibly identify the missing enzymes of the endogenous biochemical pathway. Anyhow, the here-provided transcriptomes of MRT still could represent a valuable background to uncover some still-unexplored enzymatic mechanisms related to CA de novo biosynthesis in blister beetles.

### Possible mechanisms preventing self-intoxication from CA in blister beetles

CA is a potent toxin binding to protein phosphatase 2A, a metabolic constituent of all eukaryote cells [58]. Then, regardless of where CA is synthesized, mechanisms to cope with its toxicity should exist in the accumulating MAG, but, in general, in tissues and organs that interact with this terpene. In insects, toxicokinetic detoxification of lipophilic xenobiotics is typically achieved in two phases: i) Phase I, consisting in oxidation-reduction reactions converting the xenobiotic into a polar compound and generally performed by Cyp450s, carboxylesterases, alcohol/aldehyde dehydrogenases, hydroxylases and peroxidases, and ii) Phase II, consisting in the subsequent conjugation of the (polar) degradation product with an endogenous compound (sugars, amino acids, or glutathione) usually accomplished by glucosyl-transferases, acyl-transferases and glutathione S-transferases (GST) [59–61]. Logically, it would not be beneficial for blister beetles to activate Phase I and Phase II detoxification strategies in CA storing organs, because that would alter or inactivate a de novo synthetized and advantageous terpene. Consistently, although Cyp450s and other Phase I and II enzyme-related domains were abundant in whole transcriptomes of both LT and MV (Fig. 1A) - as expected in phytophagous insects protecting themselves against the detrimental effects of secondary metabolites of plants [62] - these were not significantly over-represented in MRT (Additional file 4: Table S11-S12). Then, other self-protective strategies are more likely than direct detoxification, such as sequestration of toxic compounds in intracellular compartments (Phase III), an additional mechanism which several insects evolved to tolerate xenobiotics [63–65]. Indeed, the observation of numerous replenished vesicles inside the cells of the glandular epithelia of MAG and *vasa deferentia* of *M. proscarabeus* [47] could support a compartmentalization strategy of CA.

Phase III is operated in many organisms by membrane transport proteins belonging to ATP-binding cassette (ABC) transporters or solute carrier proteins of the Major Facilitator Superfamily (MFS) [66, 67]. ABC transporters are amongst the largest carrier families present in many phyla [68] and known to be involved in detoxification in insects [69], whereas only recently MFS have been demonstrated to mediate xenobiotic resistance in arthropods [70–72]. Again, in phytophagous insects, proteins of both families can prevent the interference of chemical plant defences with physiological processes [46, 73]. The inspection of LT and MV orthologous unigenes upregulated in MRT allowed detecting some conserved transcripts encoding ABC and MFS proteins, as well as members of other protein families involved in the mobilization of small solutes (Fig. 4A; Additional file 5: Table S16). These transporters might function in the loading of MAG or other secretory reproductive tissues with seminal fluid components (e.g. prostaglandins, lipids, peptides, hormones) [74, 75]. Along with transportation of seminal fluid substances, some of these proteins could have also evolved the ability in blister beetles to sequester and concentrate CA (or noxious CA precursors) into the vesicles of MAG to ensure protection of MRT. More likely, to prevent damages, ABC or MFS proteins could shuttle into MAG a chemically deactivated form of CA carried by the haemolymph, such as, for example, an endogenously produced glycosylated intermediate. Alternatively, the inward transportation of a diet-acquired glycosyl donor from haemolymph could serve to deactivate a noxious CA precursor in MRT. Noteworthy, both the complete absence of a cuticular partitioning and the labyrinthine lacunar system observed in the third MAG pair of *Meloe proscarabeus* [47] might support the absorption and vehiculation of substances from the haemolymph into blister beetle MRT.

Among LT and MV orthologous transporters upregulated in MRT, the proton-coupled folate transporter, sugar transporter SWEET1, sodium-coupled monocarboxylate and choline transporter-like protein 1 (Fig. 4A-B) echoed the panel of proteins governing the sequestration of the plant-derived phenolic glycoside salicin in larvae of *Chrysomela populi* [76]. By feeding on plants, larvae of this leaf beetle sequester salicin that reaches a specialized closed and chitin-coated defensive glandular reservoir via the haemolymph. In larval defensive glands the acquired glycoside is then enzymatically converted into a biologically active form, the salicylaldehyde, which is emitted as a deterrent [77–79]. Actually, the enzymatic conversion of a glycosidic precursor gathered directly from plants is considered a recent novelty evolved in leaf beetles to release toxins in a safe manner. In more ancestral leaf beetle groups, such as in *Phaedon cochleariae* Fabricius, 1791 and *Gastrophysa viridula* De Geer, 1775, monoterpenoids (iridoids) are de novo synthetized [80–82] through the conversion of an autonomously-produced 8-hydroxygeraniol-8-O-*β*-d-glucoside into the iridoids in the glandular reservoir, where cleavage and toxin release occur [81]. Likewise, to readily avoid the hazardous effects of an endogenously produced toxic terpene, glycosylation of a CA precursor should occur autonomously in blister beetles, without having to rely on sequestering compounds from diet. Therefore we hypothesize that, in order to circumvent the auto-intoxicative effects of CA, blister beetles might shuttle autonomously-synthetized CA glycosylated precursors from haemolymph into MAG. Remarkably, the enrichment in both species of glycosyl transferase domains in the transcriptomic repertoire of haemolymph (i.e. pf03360 in LT and Pf04666 in MV, Additional file 4: Table S13-S14) could support the transfer of a saccharide moiety to a circulating CA precursor.

Five other unigenes of ABC and MFS transporters which might play a role in sequestration mechanisms were identified using a more ‘relaxed’ threshold (i.e. FDR < 0.01) (Fig. 4A,C). Specifically, three of them (PLT4, l(2)03659, ABCF2) were significantly upregulated in MRT as compared to whole body (Fig. 4C) and, then, deserve further attention to understand their role in detoxification.

Whatever the biochemical mechanism to prevent damages in the storing organs, the auto haemorrhage response requires a rapid release of CA in the haemolymph to reach the intersegmental membranes of blister beetle appendages and be discharged externally. Then, once the behavioural response is triggered, a mode of transport in the haemolymph capable of mitigating the toxicity of a released and free-circulating CA in beetle tissues should also be expected. Among the proposed carriers, lipocalins have been regarded as potential CA-binding proteins able to simultaneously carry out roles related to both transport and detoxification in blister beetle haemolymph [21, 22]. Insect lipocalins bind a variety of lipophilic (endogenous or exogenous) compounds and are involved in many functions, among which olfaction, pheromone transport and metabolite (e.g. retinoic acid) binding [83]. Among the three LT and MV orthologous lipid-binding proteins upregulated in haemolymph, one was related to a phorbol ester/diacylglycerol binding protein unc-13 (whose possible role is discussed in the next paragraph), but two were, as previously hypothesized [21], associated to lipocalins (Fig. 4A). We specifically identified: (i) a possible homolog of apolipoprotein D (ApoD) (Fig. 4A), a lipocalin associated with high-density lipoproteins playing fundamental roles in lipid metabolism or in binding for progesterone and arachidonic acid in humans [84] and, (ii) a low-density lipoprotein receptor (MALRD1) (particularly abundant in LT haemolymph) able to bind to ligands (such as lipocalins) and internalize them through receptor-mediated endocytosis [85]. Among these two, ApoD was previously found transcriptionally induced in *T. castaneum* in response to the injection of crude lipopolysaccharide [86] or upon septic injury [87], thus suggesting a role of this protein in both immunity and stress responses in tenebrionid beetles. Hence, under stressful conditions, ApoD could be strongly upregulated in haemolymph of blister beetles. Once there, this protein could bind the released CA and deliver it outward during the autohaemorrhaging exudation. Since substrate binding abilities of lipocalins are broad [83], more tailored approaches are needed to confirm this hypothesis.

### Haemolymph-specific transcriptomes and possible molecular adaptation to reflex-bleeding

Blister beetles, if stressed, exude viscous-oily droplets of haemolymph from the autohaemorrhaging tissues, and this represents the manner they externally emit CA.

The autohaemorrhaging behaviour requires efficient coagulation and integument repairing mechanisms to minimize haemolymph loss [5]. Moreover, wounds in the intersegmental membranes provoked by increased haemolymph pressure are exposed to the attack of pathogens, which challenge the immunity system.

In insects the haemostatic system serves to stop bleeding, prevent microbial entry and favour wound sealing and healing. The first step in clotting formation in arthropods is achieved by the release of structural clot components following haemocyte degranulation. Among the most represented motifs in LT and MV haemolymph proteins (Table 5), we identified Von Willebrand Factors (vWF), C8 and trypsin inhibitor-like cysteine rich domains, typical of structural clotting proteins of arthropods [88, 89]. We also retrieved several transcripts encoding haemocytins - multidomain insect humoral lectins homologous to the mammalian vWF [90, 91] - owning vWF, C8 and trypsin inhibitor-like cysteine rich domains (Table 5) and that likely represent the main structural glycoproteins involved in coagulation of blister beetle haemolymph. We also found many transcripts related to calcium-dependent transglutaminases (i.e. haemocyte protein-glutamine gamma-glutamyltransferase) and prophenoloxidases (PPO), two protein classes known to cross-link the structural components in insects to harden the clot [88, 89]. The PPO-activating system constitutes an important component of insect defence mechanisms [92]: in fact, PPO released by haemocytes via a proteolytic cascade promotes the oxidation of phenolic molecules to produce melanin around invading pathogens and wounds [88, 89, 93]. We also observed other factors possibly engaged in coagulation and innate immunity, among which Spätzle and Laminin. Spätzle is a structural homolog of coagulogen, a clotting protein and functional equivalent of fibrinogen from horseshoe crab [94], but also a key regulator of the Toll pathway, leading to the expression of genes involved in immune defence to gram-positive bacteria and fungi [95]. Laminins are extracellular matrix glycoproteins of the basal lamina [96] but were demonstrated to interact with invading parasites and to play a dual role in immunity by both maintaining the basal levels of complement in the haemolymph and promoting the production of complement components through the interaction with LRIM1, as demonstrated in *Anopheles gambiae* Giles, 1902 [97].

Filamin, fascin, mucin and vinculin were among the most abundant domains in LT and MV haemolymph (Table 5; Additional file 4: Table S13-S14). Since these domains are typical of actin-bundling proteins producing gelation factors [98, 99], they could be responsible for the emission of the exuded haemolymph in the form of a mutable (liquid to solid) viscous-elastic oily substance in blister beetles. However, we cannot exclude that such factors might somehow be involved in tissue morphogenesis and repair following the autohaemorrhaging damage [100]. These repairing functions could also be performed by thymosines, whose domains were highly represented in the haemolymph of the two analysed species (Table 5). Thymosin β4, in particular, is associated with tissue repair and cell migration in vertebrates [101], albeit still scarcely characterized in insects [102]. Thymosines are also known to regulate haemopoietic stem cell proliferation and differentiation in crustaceans [103]. Indeed, haematopoiesis is expected to be enhanced after injury-induced haemolymph loss and nonself challenge [104] and an increased level of mitosis is also likely to occur within the hematopoietic tissue by immune stimulation [105]. Coherently, we found many domains related to mitosis (Table 5), such as mini-chromosome maintenance (MCM) proteins involved in chromosome replication.

We also detected some over-represented protein domains related to the diacylglycerol (DAG) /phosphatidic acid (PA) signalling pathway [106], i.e. diacylglycerol kinases (RalGDS/AF-6 and C1 domains), pleckstrin homology (PH) and PDZ-related proteins (Table 5; Additional file 4: Table S13-S14). The high representation of diacylglycerol kinases (and the upregulation of inositol 1,4,5 triphosphate phosphatase-related transcripts; Additional file 2: Table S4, S6) could indicate an active conversion of DAG to PA [107], the latter being the main substrate of two major constituents of cell membranes in eukaryotes, i.e. phosphatidylcholine and phosphatidylethanolamine [108]. PA is also known to activate the phosphoinositide signalling pathway [109] regulating many cell activities through the direct interaction of phosphoinositides with membrane proteins or by membrane recruitment of cytosolic proteins containing e.g. PH and PDZ domains able to bind phosphoinositides [110, 111]. Phosphoinositide signalling triggers cell proliferation and survival, but also induces cytoskeletal changes and actin remodelling for vesicle trafficking, membrane dynamics (and ruffling), cell division/cytokinesis and migration [112]. Hence, among all possible roles of DAG/PA signalling pathway, the increase of PA and the activation of phosphoinositide-induced processes in our target species could respond to the need of repairing cells and remodelling tissues damaged after autohaemorrhaging. These processes could also be sustained by the activity of multiple Rap/Rho GAP and Rho GEF-containing proteins (Table 5; Additional file 4: Table S13-S14), known to regulate cytoskeletal rearrangements necessary for cell-shape change, cell adhesion and migration [113]. More tailored investigations, however, are needed to clarify if the activation of the above-mentioned signalling pathways could have any role in blister beetle autohaemorrhaging response.

Finally, we observed several proteins exhibiting multiple C2 domains (Table 5). These Ca^2+^-dependent cysteine-rich modules, binding phospholipids and targeting cell membranes, are involved in several signal transduction cascades or membrane trafficking [114]. In LT and MV haemolymph C2 domains were mostly represented by transcripts annotated as Multiple C2 domain/Transmembrane region Proteins (i.e. MCTP, containing three consecutive C2 and a peroxin-like domain), synaptotagmin-1, phorbol ester/diacylglycerol binding protein unc-13 homolog B and Protein Kinase C. The exact homology and function of these transcripts need to be verified, since the presence of multiple-C2 domain proteins, synaptotagmin-1 and unc-13 homologs in insect haemolymph is rather unusual. In fact, in invertebrates these molecules are expressed in synaptic vesicles of the nervous system, where they regulate the secretion of neurotransmitters [115, 116]. Noteworthily, MCTPs were found expressed in the accessory cells of the olfactory organs of *Drosophila* and supposed to be secreted into the sensillum lymph that surrounds the olfactory receptor neuron dendrites [117]. If further analyses will confirm the homology of the retrieved transcripts with proteins involved in neuronal functions, then circulating MCTP, synaptotagmin-1 and unc-13 could neuromodulate the response to autohaemorrhaging ruptures starting from peripheral sensory organs placed in legs and antennae where these damages more often occur. These findings, if corroborated, could inform about the existence of a ‘neuronal alarm system’ allowing blister beetles to rapidly activate repairing functions and minimize haemolymph loss.

## Conclusions

This study provided a high quality de novo assembly of LT and MV male transcriptomes, a valuable genetic resource to explore some of the still enigmatic aspects of blister beetle biology. By specifically producing transcriptomes of two tissues steadily in contact with CA, i.e. the MRT and haemolymph in two species, we were able to draw some hypotheses on the mechanisms employed by these insects to guarantee a safe storage, circulation and autohaemorrhaging emission of CA. We were also able to identify for the first-time a panel of haemolymph factors expressed in the reflex-bled exudate of blister beetles. Finally, we contributed to characterize the expression levels in MRT and haemolymph of genes so-far known to be involved in the autogenous production of CA in two species never investigated thus far.

The precise mechanisms allowing CA to be accumulated in MRTs and released into the haemolymph without causing damages to internal tissues of blister beetles are still elusive. In this regard, we here propose that sequestration through ABC, MFS or solute transporters of a purported glycosylated CA precursor could avoid auto-intoxication after accumulation in MAG of this terpene. We also identified some abundantly expressed lipocalins that could vehiculate and mitigate the reactivity of a freely circulating CA in the haemolymph during the autohaemorrhaging response. Future tailored studies aimed at verifying these hypotheses and/or definitively identifying factors granting self-protection in blister beetles would shed light on one of the most intriguing aspects of their biology. Nonetheless, unveiling these mechanisms would be of great help to design innovative drug-delivery systems for a safe therapeutic application of CA in medicine. As for gene upregulation in haemolymph, we detected many factors involved in coagulation and integument repairing mechanisms in blister beetles. Their over-representation possibly reflects the need to limit the fluid loss due to the reflex-bleeding behaviour, a possible common adaptation in autohaemorrhaging insects [5] which is worth to be further analysed in an evolutionary perspective. Finally, respect to CA biosynthesis, our results are coherent with recent literature excluding a leading role of MRT in CA production at both its upstream biosynthetic steps (i.e. MVA pathway) and those related to JH catabolism. Yet, in MAG, overlooked biochemical mechanisms converting a final terpenoid intermediate into the tricyclo-decane skeleton of CA cannot be excluded. Future research efforts shall be focused to elucidate the final phases of the CA biosynthetic pathway and candidate organs where these latest enzymatic steps may occur.

## Methods

### Beetle sampling

Males of LT (tot. n. = 45, all labeled: Lazio, Roma, Tolfa, Rio Fiume, 42°03′36″N 11°56′50″E, 75 m) and of MV (tot. n. = 69; 56 labeled: Lazio, Roma, Tolfa, Piantangeli - Grasceta dei Cavallari, 42°10′52″N 11°56′30″E, 440 m; 13 labeled: Abruzzo, Pescara, Popoli, R.N. Sorgenti del Pescara, 42°10′41″N 13°48′36″E, 350 m) were collected in Central Italy in June–July 2019 by hand-picking the specimens while resting on flower stems (mainly of Dipsacaceae and Asteraceae) during the warmer hours of the day. Specimens were identified on field by the taxonomist of our research team (MAB) using taxonomic keys (Bologna 1991). Conspecific individuals were sexed by examining the last ventrite shape (deeply emarginated in males) and immediately placed in fauna-boxes with either a paper or soil bottom and provided with sand. All specimens were then transported to laboratory and kept alive for 1–2 days or, at most, a week, and fed daily with fresh flowers (e.g. *Knautia* sp.) and fruits (e.g. apple slices).

### Collection of tissues

The ‘whole body’ (or ‘total transcriptome’) reference set was constructed by immediately storing in TRIzol Reagent (Thermo Fisher Scientific, Wilmington, DE, USA) 9 males of both LT and MV (18 specimens in total). Biological replicates were obtained by sub-dividing the stored males in 3 pools, each composed by 3 individuals *per* species. Haemolymph from the remaining 36 LT and 60 MV was gathered after cutting the apical antennomers and applying a light pressure on the abdomen to facilitate the release of droplets from the cut areas. In case of poor haemolymph emission, cuts were also performed in the tarsal part of one or more legs. Haemolymph droplets were collected in ice-kept 1.5 ml Eppendorf vials filled with 1 ml TRIzol Reagent. Each of the three biological replicates of haemolymph was assembled from 12 LT and 20 MV, respectively. After haemolymph extraction, the MRT from 15 (out of 36) LT and 15 (out of 60) MV were dissected, and 3 biological replicates composed by 5 of each were generated. Each specimen was put in a sterile Petri dish and quickly dissected with scissors and tweezers under a stereomicroscope. Ventrites were removed to expose the male internal genitalia and a drop of RNase-Free water was added to facilitate the isolation of MAG from the contiguous internal organs. After their dissection, the three pairs of MAG, testicles and *vasa deferentia* were gently pulled out with tweezers and put in 1.5 ml vials (Eppendorf) kept on ice with 1 ml TRIzol.

### RNA extraction, quantification and sequencing

Total RNA from tissues was isolated using a TRIzol-based procedure (Thermo Fisher Scientific, Wilmington, DE, USA). Total bodies were grounded in a sterile ceramic mortar using liquid nitrogen to obtain a fine powder, and then homogenized in 1 ml of TRIzol Reagent. Pooled tissues of haemolymph and MRT were lysed directly in TRIzol by pipetting the lysate several times until complete homogenization. RNA concentration and purity were determined by measuring absorbance using NanoDrop 2000 Spectrophotometer (Thermo Fisher Scientific, Wilmington, DE, USA). 1 μg of total RNA was run on a 1% denaturing gel to verify RNA integrity. For each sample (‘pool’, see above) 10 μg (from total body or MRT) or 1–4 μg (from haemolymph) of high-quality RNA (RIN value > 8) was sent to IGA Technology Services s.r.l. (Udine, IT) for mRNA-seq stranded library preparation, validation, and sequencing, resulting in 9 libraries for each species. A 2 × 150 bp paired-end sequencing was performed using a NovaSeq 6000 System (Illumina, San Diego, California, USA) with a depth of about 30 million (haemolymph and MRT) or 60 million (total body) of reads *per* sample. Data generated in the present study are available in the Sequence Read Archive (SRA) database of NCBI (http://www. ncbi.nlm.nih.gov/sra) under bioproject number PRJNA674987.

### De novo transcriptome assembly, abundance estimation and differential expression analysis

Read quality was assessed by FastQC software v0.11.4 (http://www.bioinformatics.babraham.ac.uk) and read quality trimming was performed by Trimmomatic software (v0.32) [118]. The whole quality-trimmed read dataset (including reads from all tissues and body samples) for each species, concatenated into two paired FASTQ files, was de novo assembled using the Trinity software (release 2.3.2) [119, 120] with default parameters and --SS_lib_type RF, −-jaccard_clip, −-normalize_reads, −-min_contig_length 400 flags set on the ADA Server at the Department of Biology, University of Naples Federico II (24 cores, 256 GB of memory) [121]. The quality of the assembled transcriptomes was assessed by BUSCO (v3.0.1) pipeline [122]. Transcript-level quantification for each sample was performed using RSEM software [123] and Bowtie aligner [124], as implemented in the Trinity software package. Quality control between RNA-seq replicates was performed using the PtR Trinity perl script (v0.32); very good correlation scores (R^2^ > 80) were obtained for all replicates in both species, except for the third replicate from total body tissue of LT that was excluded from subsequent analyses. DGE analysis was performed using edgeR software [125], which uses a negative binomial model for differential expression analysis, with cut-off values of FDR (False Discovery Rate) < 0.001 and FC (Fold Change) > 2. FDR = *p*-value adjusted for multiple testing with the Benjamini-Hochberg procedure.

### Functional annotations

The functional categories present in the two assembled transcriptomes were investigated and summarized using Annocript pipeline (https://github.com/frankMusacchia/Annocript) [126] and UniProtKB reference database. The longest transcript of each Trinity gene cluster was employed as representative for the annotation. We performed the following similarity searches: BLASTX against TrEMBL/UniRef and SwissProt (parameters: e-value 1E-5, threshold 18, wordsize 4), RPSBLAST against CDD profiles (parameters: e-value 1E-5), BLASTn against Rfam and rRNAs (parameters: e-value 1E-5). GO (Gene Ontology) and PFAM annotations were obtained, for each transcript, using default Annocript pipeline parameters. The enrichment analysis was then performed on the GO and PFAM terms of DE transcripts, identified by edgeR in each pairwise tissue comparisons and for each species, using Annocript and the Fisher Exact Test (adjusted *p*-value < 0.01) in [R] package (www.R-project.org).

### Manual BLAST searches for genes putatively involved in CA biosynthesis

Manual searches were performed to detect unigenes that coded enzymes supposed to be involved in CA regulation in blister beetles and mapped in the ‘terpenoid backbone’ (map 00900) and ‘insect hormone’ (map 00901) biosynthetic pathways of Kyoto Encyclopedia of Genes and Genomes (KEGG; www.genome.jp/kegg). tBLASTn searches were run on LT and MV transcriptomes by using *Tribolium* (Tenebrionoidea: Tenebrionidae) or preferentially (when available) *Epicauta* or *Hycleus* (Tenebrionoidea: Meloidae) protein sequences deposited in GenBank (and reported in detail in Additional file 5: Table S15) as a query. Among transcripts showing the highest matching scores (and lowest e-values)*,* only those aligning with more than a half of residues of the query length and showing an identity percentage > 60% were considered. To confirm these results, manual BLASTn searches were also performed using the same matching threshold against LT and MV transcriptomes for the subset of genes whose mRNA sequences were available in Genbank for *Epicauta* and/or *Hycleus*. Transcripts with the highest scores and identity percentages for the searched gene (thus, representing the most plausible closest orthologs) and with FPKM> 1 for at least one of the three entries i.e. body (B), haemolymph (H) and male reproductive tract (MRT), were retained; if all variants of the “best orthologs” scored FPKM< 1, these were all reported.

### Comparative analysis of LT and MV orthologs to identify genes involved in CA deactivation and/or transport

Based on the assumption that proteins responsible for CA detoxification in the accumulating organs should be conserved across species, we optimized the selection of candidate genes by searching for putative orthologous transcripts upregulated (FDR < 0.01) in MRT versus body samples of both LT and MV. Similarly, we searched for orthologous transcripts upregulated in haemolymph versus body samples of both species to identify possible transport-related proteins. The two MRT upregulated transcript lists, and the two haemolymph upregulated transcript lists were pairwise compared using a Best Reciprocal BLASTn Hit approach with a custom Perl script and a coverage cutoff of 30% and an e-value cut-off of 0.01. To detect potential CA detoxifying enzymes or transporters, we selected common upregulated transcripts in MRT and haemolymph containing functional protein domains typical of: i) enzymes participating to the modification/degradation of apolar xenobiotics (e.g. CypP450s, carboxylesterases, hydroxylases, peroxidases, GSTs, glycosyl transferases and acyltransferase), ii) transporters involved in sequestration of terpenes/drugs and/or xenobiotics in insects (e.g. ABC-transporters, Multidrug-resistance and MFS proteins [66, 127] and iii) small solute carriers. Additionally, we searched for common upregulated transcripts in haemolymph encoding proteins previously hypothesized to bind and mobilize CA, such as lipophorins, apolipoprotein, lipocalins and General Odorant Binding Proteins [21, 22]. After manual inspection, we selected candidate orthologous transcripts from a broader list provided by the ‘Best Reciprocal Hit’ output including 483 and 283 unsorted pairs of transcripts from gonads and haemolymph, respectively. In doing so, all “unknown transcripts” which failed to receive a functional domain by the automatic annotation procedure were further scanned using InterProScan [128] and HHpred [129].

### Validation of transcripts by RT-qPCR

Total RNA was extracted from whole bodies and dissected MRT to obtain three novel replicates for RT-qPCR analysis. 1 μg of total RNA extracted from MRT and whole body samples was retro-transcribed into cDNA by SuperScript™ III Reverse Transcriptase (Thermo Fisher Scientific, Wilmington, DE, USA), following the manufacturer’s instructions. The RT-qPCR was performed using the SsoAdvanced™ Universal SYBR® Green Supermix (BIO-RAD Laboratories Inc., Hercules, CA, USA), according to manufacturer’s instructions. Amplifications were conducted for: i) β-Actin (β-ACT), Ribosomal Protein S7 (RP S7), as reference genes; ii) Juvenile hormone acid O-methyltransferase (JHAMT), Methyl farnesoate epoxidase (MFE), Juvenile hormone epoxide hydrolase (JHEH) of the JH pathway; iii) Proton-coupled folate transporter (PCFT), Sugar transporter Sweet 1 (SWEET1), Sodium monocarboxylate cotransporter 1 (SMCT1), Choline transporter-like protein 1 (SLC44A1), as potential orthologs of salicin sequestring proteins of *C. populi*); iv) Polyol transporter 4 (Polt4), Probable multidrug resistance-associated protein lethal (2)03659 (l(2)03659), Multidrug resistance protein homolog 49 (MDR49), ATP-binding cassette sub-family F member 2 (ABCF2), ATP-binding cassette sub-family B member 6 (ABCB6), as other (MFS/ABC) transporters of interest. Specific primers pairs were designed (Additional file 6: Table S17). All reactions were performed in triplicate in the Corbett Rotor-Gene 6000 (Qiagen, Hilden, Germany) and relative quantification was carried out with the ΔΔCT method [130] using the abundance of RPS7 mRNA as endogenous housekeeping control. The relative transcription levels as obtained by RT-qPCR analyses were therefore compared with abundance levels as detected by RNA-seq (FPKM, fragments per kilobase of exon model per million reads mapped - the same values employed to produce the Heat Maps in Fig. 3-4). Values from replicate experiments were averaged. Finally, to obtain values suitable for statistical comparisons, we calculated (for each gene) a fold-change (FC) value as the ratio of abundance of each transcript (ΔCt and FPKM for RT-qPCR and RNA-seq, respectively) over the group median. These values (plotted after conversion in log2 numbers) were used to evaluate the correlation between RNA-seq and RT-qPCR methods, applying statistical evaluation throughout the Pearson test (by using the Prism GraphPad software).

## Supplementary Information

**Additional file 1 Table S1-S2.** Annotation and sequence data of LT and MV longest transcript for each gene cluster.

**Additional file 2 Table S3-S6.** Annotation of upregulated transcripts in MRT and haemolymph (as compared to whole body) of LT and MV (FDR < 0.001 –logFC> 2).

**Additional file 3 Table S7-S10.** Complete list of significantly (adj. *p* < 0.05) over-represented Gene Ontology (GO) terms in differentially expressed transcripts of MRT and haemolymph of LT and MV.

**Additional file 4 Table S11-S14.** Complete list of significantly (adj. *p* < 0.05) upregulated protein families (Pfam) in MRT and haemolymph of LT and MV.

**Additional file 5 Table S15-S16.** Expression data (FPKM) and complete list of transcripts putatively involved in CA biosynthesis pathways and sequestration/self-detoxification mechanisms in LT and MV.

**Additional file 6 Table S17.** List of primers used for RT-qPCR validation.

## Data Availability

The short-read DNA sequences have been deposited in the Sequence Read Archive (SRA) database of NCBI (http://www. ncbi.nlm.nih.gov/sra) under bioproject number PRJNA674987.

## Abbreviations

ABC: ATP-binding cassette
ALP: Alkaline phosphatase
ApoD: Apolipoprotein D
atoB: acetyl-CoA C-acetyltransferase
BLAST: Basic local alignment search tool
BUSCO: Benchmarking set of universal single-copy orthologues
CA: Cantharidin
Cyp450: Cytochrome P450
DAG: Diacylglycerol
DEGs: Differentially expressed genes
FDR: False discovery rate
FDS: Farnesyl diphosphate synthase
FNTB: protein farnesyltransferase subunit beta
FOHSDR: NADP+-dependent farnesol dehydrogenase
FPKM: Fragments per kilobase million
FPP: Farnesyl diphosphate
GO: Gene Ontology
GST: Glutathione S-transferase
HMGCR: 3-hydroxy-3-methylglutary-CoA reductase
HMGCS: hydroxyl-methylglutaryl-CoA synthase
ICMT: protein-S-isoprenylcysteine O-methyltransferase
KEGG: Kyoto encyclopedia of genes and genomes
JH: Juvenile hormone
JHAMT: JH acid O-methyltransferase JH-III synthase
JHEH: Juvenile hormone epoxide hydrolase
LT: *Lydus trimaculatus*
MAG: Male accessory glands
MALRD1: MAM and LDL-receptor class A domain-containing protein
MCM: Mini-chromosome maintenance
MV: *Mylabris variabilis*
MVA: Mevalonate
mvaK1: Mevalonate kinase.
mvaK2: phosphomevalonate kinase
MFE: Methyl farnesoate epoxidase
MFS: Major Facilitator Superfamily
MCTP: Multiple C2 domain and Transmembrane region Proteins
MRT: male reproductive tract
MVD: diphosphomevalonate decarboxylase
PA: Phosphatidic acid
Pfam: Protein Family
PH: Pleckstrin homology
PPO: Prophenoloxidase
RT-qPCR: Reverse transcription-quantitative PCR
STE24: STE24 endopeptidase
Uniref: UniProt reference clusters
vWF: von Willebrand Factor
